# Impact of Telemedicine Adoption on Hemiplegia in Patients With Stroke in Florida: Longitudinal Observational Study

**DOI:** 10.2196/72315

**Published:** 2025-05-23

**Authors:** Yao Li, Mingshan Zhang, Weiling Ke, Wenwen Li

**Affiliations:** 1 Department of Information Systems and Management Engineering Southern University of Science and Technology Shenzhen China; 2 School of Management Fudan University Shanghai China

**Keywords:** telemedicine, stroke, hemiplegia, tissue plasminogen activator, longitudinal studies

## Abstract

**Background:**

Telemedicine has emerged as a critical tool in the management of acute stroke; yet, its impact on clinical decision-making, particularly in the administration of tissue plasminogen activator (tPA), remains underexplored. Understanding how telemedicine influences tPA use and subsequent patient outcomes, such as hemiplegia, is critical for optimizing stroke care protocols.

**Objective:**

This study aims to assess whether the adoption of telemedicine influences treatment decisions regarding tPA administration in patients with stroke. In addition, we used a causal mediation framework to examine the causal path between telemedicine adoption and the occurrence of hemiplegia via tPA use. Furthermore, we conducted a moderated mediation analysis to investigate the extent to which regional differences (metropolitan vs nonmetropolitan locations) impact this mediated relationship.

**Methods:**

We analyzed data of patients with stroke from Florida’s State Emergency Department Database (SEDD), State Inpatient Database (SID), and the Healthcare Information and Management Systems Society (HIMSS) database, covering the years 2010 to 2017, with a focus on telemedicine adoption. The final sample includes 314,665 visits from patients with stroke. A fixed-effects model was used to examine the relationship between telemedicine adoption and tPA use and between tPA use and hemiplegia occurrence. A causal mediation framework was then applied to estimate the average direct effect and the average causal mediation effect of telemedicine on hemiplegia through tPA use. In addition, a moderated mediation analysis was performed to explore how metropolitan status influences the mediation process.

**Results:**

We found that telemedicine adoption is associated with a 1% decrease in tPA use (coefficient=–0.010; 95% CI –0.013 to –0.007; *P*<.001), and that the use of tPA is associated with a 23.7% increased probability of hemiplegia (coefficient=0.237, 95% CI 0.231-0.243; *P*<.001). Consequently, telemedicine adoption was found to reduce the likelihood of hemiplegia by decreasing tPA usage. The causal mediation analysis shows a negative average causal mediation effect (average causal mediation effect=–0.002, 95% CI –0.003 to –0.002; *P*<.001), suggesting that telemedicine adoption reduces hemiplegia occurrence, while the average direct effect is not statistically significant (average direct effect=–0.002, 95% CI –0.007 to 0.004; *P*>.10). Importantly, the positive effect of telemedicine on reducing hemiplegia is observed only among metropolitan patients.

**Conclusions:**

This study provides evidence that telemedicine adoption can improve stroke care by reducing tPA administration, thereby lowering the risk of hemiplegia. However, the benefits appear more pronounced in metropolitan areas, highlighting potential regional disparities in stroke care. These findings underscore the importance of targeted interventions to ensure equitable access to telemedicine, especially in rural and underserved areas. Policy makers should focus on enhancing telemedicine infrastructure and training in these regions to optimize stroke care and reduce health inequities.

## Introduction

Stroke is a critical acute cerebrovascular condition with high global morbidity and mortality, significantly contributing to long-term disability and health care costs [1]. In the United States, the urgency of stroke care is underscored by the occurrence of a stroke every 40 seconds and a death every 4 minutes, making it a major public health concern [2]. Effective treatment, especially for acute ischemic stroke, demands rapid and expert evaluation to ensure timely administration of tissue plasminogen activator (tPA), a critical intervention in reducing long-term neurological deficits [2].

The clinical decision-making process surrounding tPA administration is crucial but fraught with challenges. tPA, when administered within a narrow therapeutic window of 3 hours from symptom onset (extendable to 4.5 hours for eligible patients), can significantly reduce mortality and improve functional outcomes [2,3]. However, the improper use of tPA in stroke care can lead to significant risks, including hemorrhagic stroke and the occurrence of hemiplegia [4]. These risks highlight the importance of precise, timely, and accurate clinical judgment to maximize tPA’s benefits while minimizing its potential harm [5]. The complexity of stroke treatment and the critical time window underscore the urgency of swift and accurate decision-making in stroke care.

Community hospitals often face significant challenges in providing timely stroke treatment due to limited specialized expertise and resources. This deficiency frequently results in patient transfers to academic stroke centers, which, while necessary, introduce delays, additional costs, and potential risks for patients. Moreover, the use of tPA requires careful administration, as it carries a risk of severe complications, such as intracerebral hemorrhage, which can lead to outcomes such as hemiplegia [4]. Hemiplegia, a common consequence of stroke, is often the result of extensive brain damage caused by prolonged ischemia [6].

Telemedicine has emerged as a promising solution to bridge this gap in stroke care by providing remote access to stroke specialists [2,7]. Through real-time video consultations, telemedicine allows health care providers at community hospitals to collaborate with neurologists at academic centers, enabling them to evaluate patients, interpret diagnostic imaging, and make critical therapeutic decisions, including the administration of tPA [8]. This aligns with findings from the American Heart Association, which emphasizes the role of telemedicine in standardizing stroke care protocols and expanding access to time-sensitive treatments [9]. Studies have shown that telemedicine can positively impact clinical decision-making, such as an increase in the use of thrombolytics in remote hospitals integrated into telemedicine networks [10]. However, while the relationship between telemedicine adoption and patient outcomes has been well documented, the underlying mechanisms mediating this relationship remain insufficiently understood. Moreover, despite the potential of telemedicine to reduce regional health care inequality, empirical evidence on its effectiveness remains mixed, with some studies suggesting it may alleviate disparities [11], while others indicate it might intensify them [12,13].

This study aims to empirically investigate how telemedicine adoption influences treatment decisions regarding tPA use in stroke management. Additionally, it examines the role of tPA administration in mediating the relationship between telemedicine adoption and patient outcomes, particularly the occurrence of hemiplegia. Furthermore, we explore the boundary conditions of telemedicine’s impact by examining regional disparities, a critical source of bias. Specifically, we use moderated mediation analysis to examine how regional differences (metropolitan vs nonmetropolitan) moderate the mediating effect of tPA use on the relationship between telemedicine adoption and the occurrence of hemiplegia. Using a causal mediation framework, this research seeks to clarify the causal pathways through which telemedicine affects stroke treatment and patient outcomes, offering valuable insights into its potential to enhance stroke care.

## Methods

### Data and Sample

This study uses data from Florida’s State Emergency Department Database (FL-SEDD) and State Inpatient Database (SID) provided by the Healthcare Cost and Utility Project (HCUP). The FL-SEDD and SID databases include detailed records of emergency department and inpatient visits across Florida. Patients with stroke were identified using the HCUP database and *ICD* (*International Classification of Diseases*) diagnostic codes (433X, 434X, 436 for *ICD-9* [*International Classification of Diseases, Ninth Revision*]; I63X, I64 for *ICD-10* [*International Statistical Classification of Diseases and Related Health Problems 10th Revision*]).

To measure the adoption of telemedicine from 2010 to 2017, we collected data from the Healthcare Information and Management Systems Society (HIMSS) database. We linked the HCUP and HIMSS databases using the American Hospital Association (AHA) ID, allowing a comprehensive analysis across the 2 sources. After merging the datasets, we obtained a final sample of 314,665 visits from patients with stroke.

### Measurement of Telemedicine

Telemedicine adoption was assessed using data from the HIMSS database, specifically from the HAEntityApplication table. Telemedicine adoption status is recorded under ApplicationID 143, where a status of “Live and Operational” indicates that the hospital has implemented telemedicine. Based on this classification, we created a binary variable, “tele,” where “tele=1” denotes telemedicine adoption, and “tele=0” otherwise.

### Measurement of tPA

The use of tPA was identified through patient procedure codes, following the classification outlined by Kleindorfer et al [14]. Specifically, tPA administration is recorded under *ICD-9* code 99.10 and *ICD-10* codes 3E0X317 and 3E0X17. A binary variable, “tPA_Use”, was created, where “tPA_Use=1” indicates that the patient received tPA treatment, while “tPA_Use=0” otherwise.

### Measurement of Hemiplegia

Hemiplegia was identified based on patient diagnosis codes, following the classification outlined by Roumie et al [15]. A binary variable, hemiplegia, was defined, where “hemiplegia=1” indicates the occurrence of hemiplegia during the treatment process, and “hemiplegia=0” indicates no occurrence of hemiplegia.

### Control Variables

To account for potential confounding factors, we included a set of control variables, including both patient demographics and hospital characteristics. Patient demographics include variables such as age, gender, Charlson Comorbidity Index, insurance types, and so on. Hospital characteristics include the number of registered beds and the type of the hospital (teaching hospital or not).

### Analytic Approach

We initially used an ordinary least square model to regress telemedicine adoption on tPA use and regress tPA use on the occurrence of hemiplegia, as outlined in equations 1 and 2. In equation 1, we use “tele_it_” to represent telemedicine adoption of hospital *i* at time *t*. If the hospital has adopted telemedicine, “tele_it_=1,” otherwise, “tele_it_=0.” In addition, we define “tPA_Use_ipt”_ to represent tPA use for patient *p* in hospital *i* at time *t*. To account for potential confounding factors and unobserved heterogeneity, our regression model incorporates a set of control variables, including hospital- and patient-level characteristics (Controls_ipt_), as well as hospital fixed effects (θ_i_) and time fixed effects (μ_t_).

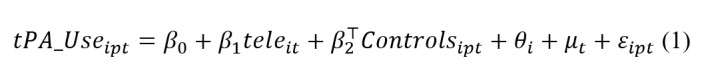



In equation 2, we regress tPA use on the occurrence of hemiplegia. The binary variable hemiplegia_ipt_ represents whether patient *p* experiences the occurrence of hemiplegia in hospital *i* at time *t*. To ensure consistency and mitigate potential confounding effects, we use the same set of control variables as in equation 1.

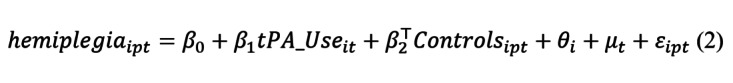



### Causality

To establish a causal relationship, we further apply a causal mediation framework, using the R package (R Foundation for Statistical Computing) “mediation” for analysis, a commonly used method for identifying causal paths in observational studies in the medical field [16-19]. Unlike traditional regression methods, the causal mediation analysis framework offers several advantages in understanding causal relationships. First, it provides a clear and consistent definition of causal effects within the potential outcomes framework, which is discussed in greater detail below [20,21]. Second, it statistically demonstrates the sequential ignorability assumption, which is crucial for causal identification [20]: (1) treatment assignment is independent of potential outcomes (both direct and mediated), given the covariates; (2) mediators are independent of potential outcomes, given both the treatment and covariates; (3) the framework offers significant flexibility in modeling various types of outcomes, including linear models, probit models, survival analysis models, and others [16,20]; and (4) finally, it incorporates sensitivity analysis to assess the robustness of the sequential ignorability assumption, ensuring the reliability of the causal inference [20].

In this context, causal mediation analysis can estimate the average direct effect (ADE) and the average causal mediation effect (ACME) of the effect of telemedicine on hemiplegia through tPA use [20,21]. Specifically, the ACME captures the extent to which telemedicine adoption affects hemiplegia occurrence through tPA use, quantifying the mediation role of tPA administration in the causal pathway. The ADE represents the direct effect of telemedicine adoption on hemiplegia occurrence, accounting for any potential pathways through which telemedicine impacts hemiplegia occurrence beyond tPA administration. In the potential outcomes framework, if the hospital patient *i* visits has adopted telemedicine, the observed mediator (tPA use) under the treatment condition *T=1* is denoted as *M*_i_(1), which further influences the observed outcome *Y*_i_(1,*M*_i_(1)). Then, the potential outcome (under the treatment condition *T*=1) without telemedicine is represented as *Y*_i_(0,*M*_i_(0)). For *T*=0, the opposite occurs for the observed mediator *M*_i_(0) and the outcome *Y*_i_(0,*M*_i_(0)). For both *T*=1 and *T*=0, the total effect of telemedicine adoption on the occurrence of hemiplegia for the patient can be calculated as follows:

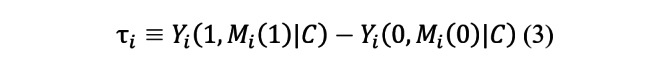



where *C* represents the same control variables that were included in equations 1 and 2. Under the potential outcomes framework [20,21], the total effect can be decomposed into the ACME (_i_(*T*)) and the ADE (_i_ (*T*)):

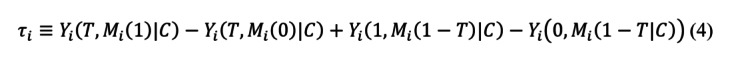

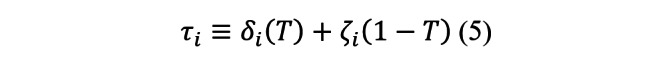





Furthermore, we conducted a moderated mediation analysis, which allows for the investigation of how certain variables (metropolitan patients in our study) may influence the mediation process. To incorporate moderating effects into the model, we introduce the moderator , which influences both the mediation and direct paths. The moderated mediation model can be expressed as follows:

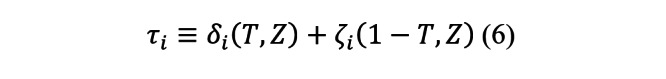



where and represent the ACME and ADE moderated by Z, respectively. In this framework, the moderator can take values of 0 or 1, allowing us to examine how the moderator influences both the mediated and direct effects of the treatment. The coefficients and CIs of ACME and ADE are estimated using a nonparametric bootstrap method [21]. To ensure robust statistical inference, we performed bootstrapping 1000 times in this study.

### Ethical Considerations

This research does not involve human subjects or human biological materials (eg, human embryos, fetuses, fetal tissue, reproductive materials, and stem cells). This research used data available in the public domain and included only organizational-level data associated with hospitals in the United States. According to Section 8 of the HCUP Data Use Agreement [22], under the HIPAA (Health Insurance Portability and Accountability Act) Privacy Rule, the use of limited datasets does not require review by an institutional review board.

## Results

### Summary Statistics

Table 1 presents the variable definitions and summary statistics for the sample of 314,665 visits by patients with stroke from 2010 to 2017. Of the hospitals in the sample, 30% have adopted telemedicine, as indicated by a mean value of 0.30 (SD 0.46). Regarding patient treatment, only 6% of patients used tPA on average (SD 0.24). On average, hemiplegia was present in 30% of cases (SD 0.46). The average number of registered beds in hospitals was 452.73 (SD 323.93), indicating varying hospital sizes. Patient demographics reveal an average age of 70.56 (SD 15) years, with a balanced sex distribution (female mean 0.50, SD 0.50). The average Charlson Comorbidity Index score is 3.16 (SD 2.22), suggesting a moderate level of comorbidities among patients. Geographically, most patients reside in metropolitan areas (mean 0.93, SD 0.26), and the majority have Medicare insurance coverage (mean 0.68, SD 0.47).

**Table 1:** Variable definitions and summary statistics.

Variables	Number	Mean (SD)	Description
Tele^a^	314,665	0.30 (0.46)	^Equal to 1 if the hospital has adopted telemedicine, otherwise equal to 0^
tPA^b^_Use	314,665	0.06 (0.24)	^The variable is assigned a value of 1 if the patient received tPA injections as part of their treatment; otherwise, it is set to 0^
Hemiplegia	314,665	0.30 (0.46)	^The variable indicates the presence of hemiplegia, with a value of 1 representing its occurrence and 0 indicating its absence^
Log(nofbed)^c^	314,665	5.87 (0.74)	^The log transformation^ ^of^ ^the number of registered beds in the hospital^
Academic	314,665	0.05 (0.21)	^Equal to 1 if the hospital is a teaching hospital, otherwise equal to 0^
Age	314,665	70.56 (15)	^Age of the patient^
Female	314,665	0.5 (0.5)	^Equal to 1 if the patient is female, otherwise equal to 0^
charlson_index^d^	314,665	3.16 (2.22)	^Charlson Comorbidity Index of the patient^
White	314,665	0.66 (0.47)	^Equal to 1 if the patient is White, otherwise equal to 0^
Black	314,665	0.18 (0.38)	^Equal to 1 if the patient is Black, otherwise equal to 0^
Micropolitan	314,665	0.04 (0.19)	^Equal to 1 if the patient is from a micropolitan area, otherwise equal to 0^
Metropolitan	314,665	0.93 (0.26)	^Equal to 1 if the patient is from metropolitan area, otherwise equal to 0^
Medicare	314,665	0.68 (0.47)	^Equal to 1 if the patient’s insurance is Medicare, otherwise equal to 0^
Medicaid	314,665	0.08 (0.27)	^Equal to 1 if the patient’s insurance is Medicaid, otherwise equal to 0^

^a^Tele: telemedicine.

^b^tPA: tissue plasminogen activator.

^c^Log(nofbed): log transformation of the number of registered beds in the hospital.

^d^charlson_index: Charlson Comorbidity Index.

### Impact of Telemedicine Adoption on tPA

Table 2 summarizes the results for the impact of telemedicine adoption on tPA use and hemiplegia. In column 2 of Table 2, our regression results indicate that telemedicine is associated with a 1% decrease in tPA use, with a statistically significant *P* value of .001 and a 95% CI of –0.013 to –0.007, after controlling for patient demographics, hospital characteristics, and time and hospital fixed effects. This finding suggests that telemedicine adoption leads to a more cautious approach to tPA use, particularly in situations where specialists from other hospitals provide remote consultations, leading to a decrease in the use of tPA.

**Table 2:** Impact of telemedicine adoption on tPA^a^ use and hemiplegia. Robust SEs are clustered at the patient level.

Variable	tPA_Use	tPA_Use	Hemiplegia			
	(1)	(2)	(3)	(4)	(5)	(6)
Tele^b^, regression coefficient (SE)	–0.004^c^ (0.001)	–0.010^c^ (0.001)	—^d^	–0.013^c^ (0.002)	—	–0.002 (0.003)
tPA_Use, regression coefficient (SE)	—	—	0.240^c^ (0.003)	0.240^c^ (0.003)	0.237^c^ (0.003)	0.237^c^ (0.003)
Age, regression coefficient (SE)	–0^c^ (0)	–0^c^ (0)	0.001^c^ (0)	0.001^c^ (0)	0.001^c^ (0)	0.001^c^ (0)
Female, regression coefficient (SE)	0.001 (0.001)	0.002^e^ (0.001)	0.024^c^ (0.001)	0.024^c^ (0.001)	0.026^c^ (0.001)	0.026^c^ (0.001)
charlson_index^f^, regression coefficient (SE)	0.005^c^ (0)	0.005^c^ (0)	0.081^c^ (0)	0.081^c^ (0)	0.079^c^ (0)	0.079^c^ (0)
White, regression coefficient (SE)	0.007^c^ (0.001)	0.007^c^ (0.001)	–0.011^c^ (0.002)	–0.011^c^ (0.002)	–0.020^c^ (0.002)	–0.020^c^ (0.002)
Black, regression coefficient (SE)	–0.015^c^ (0.001)	–0.014^c^ (0.002)	0.017^c^ (0.003)	0.011^c^ (0.003)	0.004 (0.003)	0.004 (0.003)
Medicare, regression coefficient (SE)	–0.006^c^ (0.001)	–0.006^c^ (0.001)	–0.019^c^ (0.002)	–0.019^c^ (0.002)	–0.020^c^ (0.002)	–0.020^c^ (0.002)
Medicaid, regression coefficient (SE)	–0.008^c^ (0.002)	–0.009^c^ (0.002)	0.032^c^ (0.003)	0.032^c^ (0.003)	0.030^c^ (0.003)	0.030^c^ (0.003)
Micropolitan, regression coefficient (SE)	–0.008^g^ (0.003)	–0.005 (0.004)	–0.002 (0.005)	–0.001 (0.005)	0.012^h^ (0.006)	0.011^h^ (0.006)
Metropolitan, regression coefficient (SE)	0.004^h^ (0.003)	–0.009^c^ (0.003)	–0.017 (0.004)	–0.003 (0.004)	–0.014^c^ (0.005)	–0.014^g^ (0.005)
Log(nofbed)^i^, regression coefficient (SE)	0.021^c^ (0.001)	–0.028^c^ (0.006)	0.012^c^ (0.001)	0.013^c^ (0.001)	0.008 (0.009)	0.008 (0.009)
Time_FE^j^	Yes	Yes	Yes	Yes	Yes	Yes
Hospital_FE	No	Yes	No	No	Yes	Yes
N	314,665	314,665	314,665	314,665	314,665	314,665
*R* ^2k^	0.010	0.020	0.182	0.183	0.199	0.199

^a^tPA: tissue plasminogen activator.

^b^Tele: telemedicine.

^c^*P<.*001.

^d^Not applicable.

^d^*P<.*05.

^f^charlson_index: Charlson Comorbidity Index.

^g^*P<.*01.

^h^*P<.*10.

^i^Log(nofbed): log transformation of the number of registered beds in the hospital.

^j^FE: fixed effect.

^k^*R*^2^: coefficient of determination.

This decrease is likely driven by the remote specialists who might influence the decision to administrate tPA for patients who are not suitable candidates. In conclusion, our regression results show telemedicine adoption is associated with a 1% reduction in tPA use. While the magnitude of this reduction is modest, this 1% reduction in tPA use is notable given the baseline 6% rate of tPA administration.

### Impact of tPA Use on the Occurrence of Hemiplegia

Columns 3-6 of Table 2 report regression results of the association between tPA use and the incidence of hemiplegia, controlling for different sets of covariates. As shown in column 6 of Table 2, which includes the full set of control variables, we observed a positive relationship between tPA use and the occurrence of hemiplegia (coefficient=0.237, 95% CI 0.231-0.243; *P*<.001). Specifically, tPA use is associated with a 23.7% increase in the probability of hemiplegia occurrence. These findings suggest that improper use of tPA can lead to adverse effects, potentially increasing the risk of hemiplegia, which underscores the importance of careful and precise medical decision-making and clinical judgment when administering tPA.

### Causal Mediation Analysis

We present the results of causal mediation analysis in Tables 3 and 4. In Table 3, our causal mediation analysis reveals that telemedicine adoption (tele) reduces the occurrence of hemiplegia (hemiplegia) by 0.2% through decreased tPA use (tPA_Use), as indicated by a statistically significant negative ACME (ACME=–0.002, 95% CI –0.003 to –0.002; *P*<.001). Our findings suggest that telemedicine adoption acts as an intermediary in the decision-making process, guiding clinicians to make more informed choices regarding tPA use, thereby reducing the probability of committing type II errors. This relationship is mediated entirely by the reduction in tPA use, as indicated by the nonsignificant ADE between telemedicine and hemiplegia (ADE=–0.002, 95% CI –0.007 to 0.004; *P*>.10). This points to the fact that telemedicine’s primary impact lies in shaping the decision-making process around tPA administration, which subsequently influences the incidence of hemiplegia.

**Table 3:** Effect of “tele” on hemiplegia through “tPA_Use.” A total of 1000 simulations were conducted and the number of observations was 314,665.

	Estimate	95% CI	*P* value
ACME^a^	–0.00225	–0.00302 to –0.00151	<.001
ADE^b^	–0.00157	–0.00685 to 0.00369	.60
Total effect	–0.00383	–0.00919 to 0.00159	.18

^a^ACME: average causal mediation effect.

^b^ADE: average direct effect.

**Table 4:** Effect of “tele” on “hemiplegia”through “tPA_Use”: moderated mediation analysis. A total of 1000 simulations were conducted and the number of observations was 314,665.

Treatment effect	Estimate		Difference
	Nonmetropolitan	Metropolitan	
ACME^a^	0.00014	–0.00248^b^	–0.00266^c^
ADE^d^	0.00519	–0.00217	–0.00762
Total effect	0.00532	–0.00465	—^e^

^a^ACME: average causal mediation effect.

^b^*P*<.001.

^c^*P*<.05.

^d^ADE: average direct effect.

^e^Not applicable.

However, our analysis reveals that the positive effect is observed primarily for metropolitan patients, indicating that telemedicine may have different impacts depending on the regional context. In metropolitan areas, where telemedicine adoption is more prevalent and resources for remote consultations are more accessible, the benefits of telemedicine are more pronounced (ACME=–0.00248; *P*<.001). However, this same effect is not as noticeable in rural or less accessible areas (*P*>.10), suggesting that telemedicine adoption could intensify regional disparities in stroke care. These findings highlight the need for targeted interventions and additional resources in underserved areas to ensure equitable access to telemedicine-based stroke care, preventing the intensity of regional health disparities.

## Discussion

### Principal Findings

This study explores the impact of telemedicine adoption on stroke care, specifically focusing on treatment decisions regarding tPA administration and the occurrence of hemiplegia. Our findings suggest that telemedicine adoption leads to a more cautious approach to tPA use, resulting in a 1% reduction in its administration. This effect can be attributed to the input of remote specialists, who are likely to contribute to more conservative decision-making, especially when evaluating whether a patient is a suitable candidate for tPA. The reduction in tPA use is particularly meaningful given the narrow therapeutic window for tPA administration and the associated risks of improper administration.

In addition, our analysis reveals that improper use of tPA increases the risk of hemiplegia, a common and debilitating outcome of stroke. Specifically, we found that tPA use is associated with an increase in the probability of hemiplegia, which aligns with prior research suggesting that tPA may not be suitable for all patients with stroke [8,23]. The heightened risk of adverse outcomes underscores the importance of precise clinical judgment when deciding whether to administer tPA. It also highlights the critical role of telemedicine in supporting informed decision-making, especially in situations where local expertise is limited or unavailable. Moreover, the findings in our study are drawn from observational studies using large-scale patient records, offering a more comprehensive understanding compared with the smaller sample sizes used in experimental settings, such as the 222 individuals in the study by Meyer et al [23] and 498 individuals in the study by Cutting et al [8].

Furthermore, our causal mediation analysis shows that telemedicine adoption reduces the occurrence of hemiplegia by decreasing tPA use. This effect is fully mediated through the reduction in tPA administration, suggesting that telemedicine’s primary contribution lies in influencing clinical decisions rather than directly affecting patient outcomes. Notably, the benefits of telemedicine adoption are more pronounced in metropolitan areas, where access to remote consultation is more readily available. In contrast, rural and underserved regions, where telemedicine adoption is less widespread, do not experience the same positive outcomes, indicating that telemedicine may inadvertently exacerbate regional disparities in stroke care.

### Limitations

One limitation of this study is its reliance on data from a single state, Florida, which may limit the generalizability of the findings. The impact of telemedicine adoption on stroke care could vary across different states in the United States or in other countries, where health care infrastructure, policies, and practices differ. In addition, the geographic variation in telemedicine adoption presents another limitation. While our analysis shows more pronounced benefits in metropolitan areas, it may not fully account for regional disparities in health care infrastructure, patient demographics, and health care provider practices. These factors could influence the effectiveness of telemedicine in stroke care and contribute to the observed differences in outcomes.

Another key limitation of this study is its exclusive focus on tPA administration as a critical service process in stroke care. While tPA is an essential treatment, it constitutes only one component of the comprehensive care required for patients with stroke. Due to data constraints, we were unable to investigate a broader range of clinical processes that telemedicine may influence. With access to more granular clinical data, a more thorough examination of how telemedicine impacts various stages of stroke care, from initial assessment to rehabilitation, would provide a deeper understanding of its overall effect on patient outcomes.

### Conclusions

Our study provides empirical evidence that telemedicine adoption can improve stroke care by facilitating more informed clinical decisions, particularly regarding the administration of tPA. By enabling remote consultations with stroke specialists, telemedicine fosters more cautious decision-making, leading to reduced tPA use and subsequently a lower risk of hemiplegia. However, the benefits of telemedicine are not uniformly experienced across regions. The positive effects are more pronounced in metropolitan areas, suggesting that telemedicine adoption could inadvertently exacerbate regional disparities in stroke care.

These findings underscore the potential of telemedicine in improving stroke management, but they also highlight the need for targeted interventions to ensure equitable access to telemedicine technologies. Specifically, rural and underserved regions must be prioritized to ensure they can fully benefit from telemedicine innovations. Policymakers and health care providers should focus on strategies that enhance telemedicine infrastructure and training in these areas, to maximize the effectiveness of telemedicine and reduce health inequities. Future research should aim to tailor telemedicine interventions to local contexts and investigate the long-term effects of telemedicine adoption on patient outcomes in stroke care.

## Abbreviations

ACME: average causal mediation effect
ADE: average direct effect
AHA: American Hospital Association
FL-SEDD: Florida State Emergency Department Database
HCUP: Healthcare Cost and Utility Project
HIMSS: Healthcare Information and Management Systems Society
HIPAA: Health Insurance Portability and Accountability Act
ICD: International Classification of Diseases
ICD-9: International Classification of Diseases, 9th Revision
ICD-10: International Statistical Classification of Diseases and Related Health Problems 10th Revision
SID: State Inpatient Database
tPA: tissue plasminogen activator

## Data Availability

The datasets used in this study are publicly available from the Healthcare Cost and Utility Project [24] and the Healthcare Information and Management Systems Society [25].
